# Developing a Shared Understanding of Humanism in Health Care Through Critical Review and Empirical Exploration: Protocol for a Modified e-Delphi Study

**DOI:** 10.2196/86055

**Published:** 2026-09-02

**Authors:** Diane Guay, Annie Turcotte, Marie-Josée April, Véronique Foley, Jacinthe Beauchamp, Nicole Marquis, Michèle Héon-Lepage

**Affiliations:** 1 School of Nursing Faculty of Medicine and Health Sciences Université de Sherbrooke Longueuil, QC Canada; 2 Faculty of Medicine and Health Sciences Université de Sherbrooke Sherbrooke, QC Canada; 3 School of Rehabilitation Faculty of Medicine and Health Sciences Université de Sherbrooke Sherbrooke, QC Canada; 4 Department of Family and Emergency Medicine Faculty of Medicine and Health Sciences Université de Sherbrooke Moncton, NB Canada; 5 Patient-Partner Initiative Faculty of Medicine and Health Sciences Université de Sherbrooke Sherbrooke, QC Canada

**Keywords:** humanism in health care, definitions, statements, medical education, modified e-Delphi study, critical review, health care professional, interdisciplinarity, qualitative analysis

## Abstract

**Background:**

Humanism is central to the practice of health care professionals but remains poorly defined and inconsistently applied. Amid growing system pressures and dehumanization, this study aims to develop consensus-based conceptual and operational definitions of humanism to support education, evaluation, practice, and policy.

**Objective:**

This study aims to formulate consensus-based conceptual and operational definitions of humanism in health care, based on the integration of theoretical perspectives with the experiences and expertise of clinician-educators, students, and members of the public.

**Methods:**

A 2-phase mixed methods design was implemented to ensure methodological rigor and analytical depth. In phase I, a critical review of the literature was conducted and systematically analyzed using content analysis. Concurrently, focus groups were conducted with learners, clinician-educators, and members of the public. The resulting qualitative data were subjected to thematic analysis to capture both the theoretical and experiential dimensions of humanism in health care. Phase 2 will involve a modified e-Delphi technique, in which the generated statements will be submitted to an interdisciplinary and cross-sectoral panel of experts for iterative refinement and validation. This approach is intended to strengthen construct validity and support consensus building.

**Results:**

This project is funded by the Research Chair in Compassion Science at the *Université de Sherbrooke*, with additional support from the Office of Social Accountability. The study is currently progressing through phase 1. In step 1 (critical literature review), the initial screening of studies based on titles and abstracts has been completed, and full-text screening is nearing completion. To date, 51 studies have been selected, and data extraction and analysis are underway. In step 2, analysis of data collected from 3 focus groups (N=18) has been completed. The process of converting critical review and focus group findings into Delphi statements is expected to be completed by the end of summer 2026. Phase 2 will consist of a modified e-Delphi process. The launch of this phase, involving the submission of the developed statements to an interdisciplinary and cross-sectoral panel of experts, is scheduled for fall 2026.

**Conclusions:**

This study is expected to contribute to the development of a conceptually grounded and operationally relevant definition of humanism in health care, informed by both theoretical foundations and lived experiences. This process will support the harmonization of educational practices, the development of assessment tools, and the integration of humanistic values into health policy.

**International Registered Report Identifier (IRRID):**

DERR1-10.2196/86055

## Introduction

Emerging from a philosophical movement dating back to the Renaissance, humanism is theoretically defined as a vision of the world centered on the value and dignity of human beings, their freedom, their responsibility, and their capacity for transformation. This common core is divided into many historical currents (scientific, critical, educational, neohumanist, transhumanist, and posthumanist contexts), which serve as a normative framework for judging social forms of life, science, and technology. Although acknowledging the plurality of humanism in contemporary literature, this study adopts a health-focused, applied definition commonly used in medical and health sciences education. From this perspective, humanism is understood as a value-based and relational orientation that places the dignity, well-being, and holistic experience of patients at the center of care rather than being viewed as an abstract or purely historical philosophical movement [1-3]. In the context of rapidly changing systems, marked by the growing integration of technology, the standardization of practices, and the increased pressure on performance, we are witnessing a silent yet profound crisis of dehumanization in health care [4]. This crisis contributes to a pronounced loss of meaning among health care professionals [1,5,6], as reflected in ever-increasing rates of burnout [7-9]. This issue affects not only the upcoming generation of physicians, nurses, and other health care professionals, who often struggle to maintain empathy and compassion over time [1,3,8,10,11], but also patients, who increasingly seek greater respect, attention, and acknowledgment [12]. Empathy and compassion are thus often relegated to the background [4] and unevenly integrated into clinical practice, training, and health policy [8].

Recent scientific literature highlights the urgent need to clarify, define, and harmonize the concept of humanism in the field of health care [4,6,8,13] to ensure human-centered care by all health care professionals [5]. It is argued that a shared understanding of humanism—from theoretical definitions to operational definitions—would improve cohesion among health care training, professional practice, and interdisciplinary collaboration [4,6,8,14-17], as well as promote the development of health policies based on common values. Without clear conceptual and operational definitions, humanism remains an abstract ideal that is difficult to model, learn, evaluate, and embody. Therefore, defining humanism in health care is not merely a semantic exercise but also an ethical, educational, and organizational imperative. Clarifying this concept is essential to preserving humanistic health care in a sustainable manner, supporting caregivers, and meeting patients’ legitimate needs and expectations.

The purpose of this study is to formulate consensus-based conceptual and operational definitions of humanism in health care, drawing on the experience and expertise of clinician-educators, students, and members of the public. Achieving conceptual clarity through this study is an essential first step toward harmonizing teaching practices within higher education institutions and standardizing clinical supervision, particularly with regard to the teaching and assessment of competencies related to humanistic care among trainees.

## Methods

### Overview

To achieve its objective of developing consensus-based conceptual and operational definitions of humanism in health care, this study adopts a structured, stepwise methodology. Guided by the DELPHISTAR (Delphi studies in social and health sciences—recommendations for an interdisciplinary standardized reporting) framework [18], this study aims to ensure methodological rigor, facilitate critical appraisal, and improve comparability with other Delphi studies in the health and social sciences, while maintaining sufficient flexibility to address the specific objectives and epistemological orientation of the research. The process unfolds in 2 main phases: an initial exploratory phase combining a critical literature review with qualitative data collection through focus groups, followed by a consensus-building phase using a modified e-Delphi technique. The critical review serves as the foundation for establishing a theoretically grounded understanding of humanism in health care, identifying conceptual gaps, tensions, and areas of inconsistency in the existing literature. The focus groups complement the review by grounding the concept in lived educational and clinical experiences, ensuring that emerging definitions reflect practice-based perspectives and contextual realities. The second phase builds directly on the insights generated in the first phase, synthesizing theoretical and empirical findings into a set of draft statements to be submitted to a modified e-Delphi process for iterative refinement and validation through expert consensus.

### Phase 1

#### Overview

**Figure 1:**
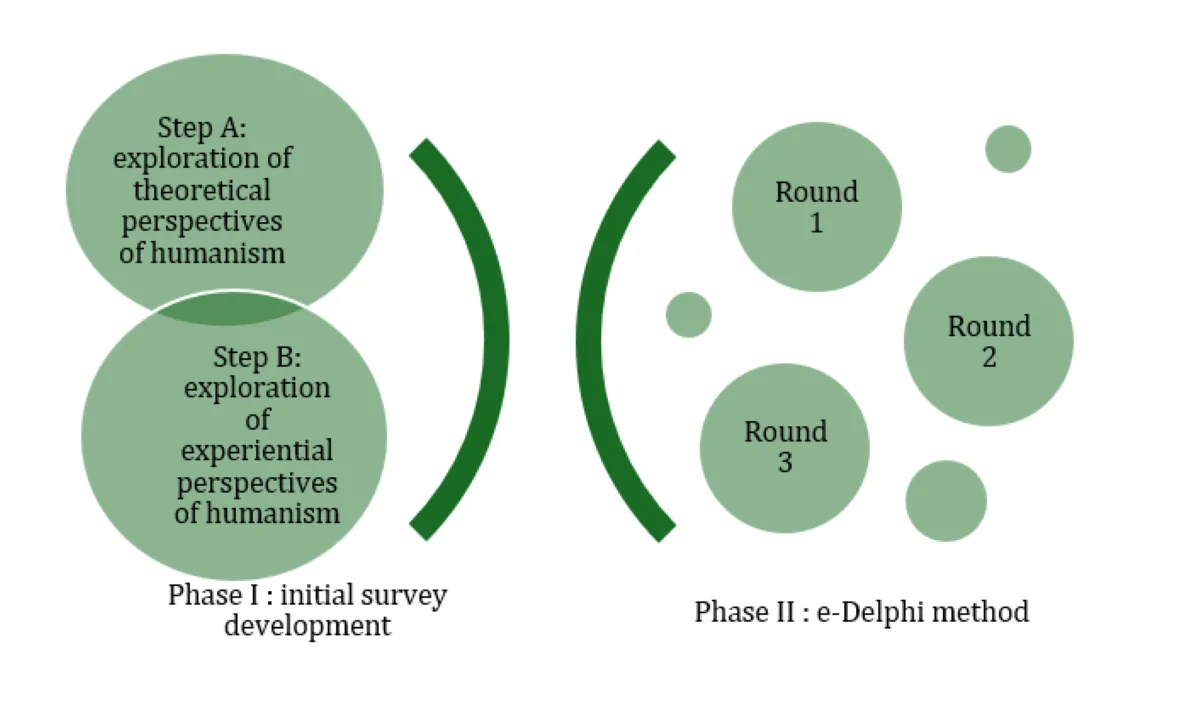
Study summary.

This phase aims to identify the statements to be included in the initial version of the survey. To achieve this objective, two complementary strategies are used: (1) a critical review of the literature on theoretical perspectives of humanism and (2) an analysis of experiential perspectives related to the concept, as illustrated in Figure 1. The integration of findings from both steps will inform the development of a preliminary version of the survey, which will subsequently be submitted to an interdisciplinary and cross-sectoral panel of experts for validation.

#### Step A: Exploring Theoretical Perspectives of Humanism

##### Overview

A critical review of the literature is underway. This type of review provides an in-depth analysis of existing publications on a specific topic. Its purpose is to synthesize current knowledge; identify trends, gaps, and future research directions; and position new research within the context of prior work. Rather than merely summarizing the literature, it critically analyzes and evaluates previous studies, adopting a reasoned and reflective stance on their findings [18]. Given that the concept of “humanism” is polysemic, this approach will make it possible to identify the main concepts associated with humanism in health care and to list their theoretical and operational definitions, where applicable [19,20].

##### Data Collection and Analysis

A literature search was conducted in Academic Search Complete, CINAHL Plus, Education Source, ERIC, MEDLINE, and SocINDEX (Textbox 1). The review includes literature related to health sciences professionals, educators, and students. Inclusion criteria were restricted to peer-reviewed papers published between 2015 and 2025 in either French or English. The corpus included empirical studies (qualitative, quantitative, and mixed methods), systematic reviews, conceptual and theoretical studies, and critical essays. Sources were included if they explicitly addressed humanism and its conceptual, pedagogical, or evaluative dimensions in the health sciences. Anecdotal texts, nonscholarly publications, and works focused solely on general professional ethics without explicit reference to humanism were excluded.

**Literature search strategy developed using the population, concept, and context framework (N=1110).**

**Population: health care providers, educators, students, and health care users**

- AB ((Health* AND (Professional* or Clinic* or Pract* or Stud* or Supervis* or Teach* or Educ* or Learn* or Residenc* or Nurs* or Medic* or Occupational* or Physio* or Physic* or User*)) or Famil* or Patient*)

**Concept: definition of humanism**

- TI (Humani*) AND AB (Concept* or Defin*or Mean* or Terminology or Framework* or Measur* or Assess* or Indicator* or Instrument* or Scale* Eval* or Teach* or Questionnaire* or Approach* or Competenc*) NOT TI (Humanitarian*) NOT TI Religio* NOT AB Religio*

**Context: health care systems and health sciences education**

- AB (Health* AND (Care or System* or Pract* or Educ* or Pedag*))

**Final search strategy**

- Population AND Concept AND Context

##### Data Analysis and Synthesis

A 2-level content analysis has been initiated to systematically interpret textual data pertaining to the concept of humanism in health care [21]. Manifest content analysis focused on extracting explicit information, particularly the identification and synthesis of formal definitions of humanism reported in the academic literature. In parallel, latent content analysis aims to uncover implicit conceptual structures and attributes embedded within these theoretical formulations, including both convergent and divergent perspectives. To enhance the analytical depth of the latent analysis, the research team adopted a structured framework based on interrogative pronouns (who, what, when, where, why, how, and about what), as proposed by Levasseur et al [22] in their scoping review of vulnerability definitions. This methodological strategy facilitates the identification of underlying assumptions, contextual determinants, and relational dynamics that are often overlooked in surface-level analyses. Applied to the study of humanism, this approach allows a nuanced and contextually grounded exploration of its theoretical dimensions, thereby contributing to the refinement and operationalization of the concept within health care research and practice.

##### Screening Process

The screening process followed a structured and transparent approach aligned with the objectives of a critical review. Following literature searches conducted within the EBSCOhost databases, titles and abstracts were screened by 2 research assistants using predefined inclusion and exclusion criteria. This process was supported by an academic librarian, who contributed to refining the search strategy and clarifying eligibility criteria. As the search strategy evolved, a member of the research team validated all adjustments to ensure consistency and methodological coherence. All potentially relevant records were retained for full-text review, which was conducted by research professionals. Throughout both screening stages, cases of uncertainty or conceptual ambiguity were discussed with a senior researcher, who provided oversight and ensured alignment with the study’s conceptual framework. The screening process was managed using Rayyan systematic review software, which facilitated record organization, eligibility tracking, and transparent documentation of inclusion decisions. Consistent with the epistemological orientation of a critical review, formal interreviewer agreement statistics were not calculated. Rather, methodological rigor was ensured through iterative discussions, expert oversight, and reflexive calibration at key decision points, particularly when interpretive judgment was required.

#### Step B: Exploring Experiential Perspectives of Humanism

##### Overview

This step constitutes the experiential component of phase 1. Its objective is to identify the core theoretical concepts that define humanism, along with their operational definitions, using an inductive approach grounded in lived experiences. Specifically, the concept of humanism in health care is explored through the distinctive and complementary perspectives of 3 categories of key informants: clinician-educators, learners, and members of the public, who have directly experienced humanism in health care settings. This approach enables the conceptualization of humanism to be informed by real-world experiences, thereby enriching and contextualizing its theoretical foundations.

##### Study Population and Sampling Strategy

Three categories of key informants were invited to participate in focus groups (Textbox 2) using a convenience nonprobability sampling strategy [23].

**Eligibility and participation criteria specific to each key informant category.**

**Learners in health sciences**

- Currently enrolled in a training program within a health-related discipline offered by the Faculty of Medicine and Health Sciences (FMSS)

**Clinician-educators**

- Health care professionals affiliated with one of the FMSS programs who actively provide direct care to individuals, groups, or communities within partner institutions of
  Université de Sherbrooke

**Members of the public**

- Individuals with personal experience navigating the health care system, either as care recipients or as caregivers for someone close to them

##### Eligibility Criteria

Participant recruitment was guided by both general eligibility criteria applicable across all key informant categories and specific inclusion parameters tailored to each subgroup. The universal eligibility criteria for participation in phase 1 focus groups were as follows: participants were required to be aged ≥18 years, demonstrate sufficient proficiency in French to actively participate in focus group discussions, have access to a computer equipped with a functional camera and microphone, and be able to independently use the Teams (version 26134.1702.4747.7366; Microsoft Corp) app.

##### Recruitment

The recruitment process followed a consistent structure across the 3 key informant categories, although the intermediaries involved in facilitating outreach differed, as outlined in Textbox 3. The research team initiated recruitment by emailing selected intermediaries, inviting them to share a recruitment poster via their social media platforms (eg, Facebook [Meta Platforms Inc] and Instagram [Meta Platforms Inc]) or through direct email to their respective networks (clinician-educators, learners, or members of the public). The poster presented a brief overview of the study and included both a hyperlink and a QR code directing interested individuals to a short eligibility and interest questionnaire. Respondents who completed the questionnaire were then contacted by a member of the research team, who provided further information about the study and addressed any questions. Following this initial contact, eligible participants received an email containing a link to the electronic information and consent form, along with a sociodemographic questionnaire. They were also invited to indicate their availability for scheduled focus group sessions. Within 2 weeks of receiving the completed forms, participants received a formal invitation with the Microsoft Teams link for their assigned focus group.

**Specific recruitment strategies for each key informant category.**

**Leaners in health sciences**

- Recruitment was facilitated through student associations affiliated with each program, as well as through the Faculty of Medicine and Health Sciences (FMSS) Student Life Service.

**Clinician-educators**

- Participants were reached through program directors, who coordinated the process with the help of their administrative staff.

**Members of the public**

- Outreach process to recruit these key informants was conducted through multiple channels, in the following order of priority:
- The FMSS Office of Social Responsibility, targeting individuals who participated in the Humanism Mobilization Day held in May 2024
- The FMSS Human Simulation and Citizen Participation Program, targeting citizens engaged in its training activities
- If necessary, collaboration with community organizations, targeting their members

##### Data Collection

Two complementary data collection strategies were implemented. Focus groups served as the primary data collection method for this stage of the study and were conducted remotely and synchronously via the Microsoft Teams platform. Videoconferencing offers enhanced flexibility and accessibility, removing barriers related to geographic location and travel time [24,25]. Each focus group lasted between 80 and 100 minutes and included key informants from 3 categories: clinician-educators (n=7), learners (n=6), and members of the public (n=5). All focus groups were audiovisually recorded and cofacilitated by 2 members of the research team using a structured discussion guide developed specifically for each informant category. These guides included open-ended questions organized around the 7 interrogative pronouns (*who, what, about what, why, when, where, and how)*, as proposed by Levasseur et al [22], to capture the unique experiential dimensions of humanism from each group. Participants were invited to recall a significant experience related to humanism from their perspective as clinician-educators, learners, or members of the public. A variety of facilitation techniques—including reformulation, clarification questions, and triadic and circular questioning—were used to minimize formulation bias and promote in-depth exploration. The order and sequence of questions were adapted to the natural flow of each discussion. All recorded sessions were transcribed by a research assistant for subsequent thematic analysis.

The second strategy involved the use of a reflective journal, which consists of reflective and critical introspection aimed at documenting the subjectivity of the research team, including perceptions, assumptions, values, decisions, and interpretations. These data served a complementary function by enhancing the transparency of the research process and facilitating the establishment of links between the data collected and the analytical interpretations [26].

##### Qualitative Data Analysis

The transcripts from the focus groups were subjected to a thematic coanalysis conducted by the research team. A mixed coding strategy, combining both deductive and inductive approaches, was applied in accordance with the methodology proposed by Miles et al [27]. To ensure that the research team’s interpretation accurately reflects the content of the focus group discussions, a summary report was drafted and emailed to participants, providing them with an opportunity to offer feedback on this collective output. This triangulation of perspectives (theoretical and experiential) will facilitate the identification of statements for an initial formulation of the conceptual (ie, abstract statements describing what humanism is) and operational definition of humanism (ie, concrete statements describing how humanism manifests in health care) to be submitted to a panel of experts. Although the number of statements cannot be predetermined, they will offer semantically rich and nuanced formulations.

#### Converting Critical Review and Focus Group Findings Into Delphi Statements

The development of statements prior to the first Delphi round will follow a systematic, multistep process designed to ensure conceptual relevance, clarity, and methodological rigor:

- First, an initial pool of potential statements will be generated through a comprehensive synthesis of the scientific literature, alongside relevant conceptual and qualitative sources. This step aims to capture a broad range of perspectives and evidence aligned with the study objectives.
- Second, the preliminary statements will undergo an internal review to identify overlap, redundancy, and ambiguity. Similar or closely related statements will be merged or refined, and the remaining items will be organized into coherent thematic domains to improve structure and interpretability.
- Finally, the refined statements will be iteratively reviewed by the research team to ensure clarity, relevance, and comprehensibility. This pre-Delphi validation step aims to confirm that each statement is clearly formulated, unidimensional, and consistent with the study’s scope and objectives [28].

The first Delphi round will serve as an additional refinement stage, allowing participants to provide both quantitative ratings and qualitative feedback. On the basis of predefined criteria and expert input, statements may be revised, merged, or removed before subsequent rounds. Overall, this iterative process ensures that the Delphi exercise begins with a well-structured, conceptually robust, and clearly articulated set of statements, while maintaining flexibility for refinement through expert consensus.

### Phase 2: Modified e-Delphi Study

#### Overview

Introduced in the 1950s, the Delphi method is a widely used data-gathering strategy for developing conceptual and operational definitions in the health sciences [29]. This technique relies on an iterative process involving successive rounds of questionnaires distributed to a panel of experts, with the aim of achieving consensus on topics for which empirical evidence remains limited [30,31]. This phase represents the operational component of the study and aims to identify consensus-based statements on humanism from the perspective of interdisciplinary experts, who will be invited to prioritize statements derived from the critical review and focus groups to support the definition, teaching, and evaluation of humanism in health care. Data will be collected via an electronic questionnaire administered through the Microsoft Forms platform, using an iterative process in which experts identify priorities and work toward consensus.

#### Study Population and Sampling Strategy

To ensure a diverse range of perspectives and complementarity of expertise, a nonprobability purposive sampling strategy will be used to recruit an expert panel of approximately 20 to 25 individuals [32]. A snowball sampling method may also be used to expand the pool as needed. Expert selection will be guided by the partnership pentagram model developed by Boelen [33], which has informed the social accountability projects in Canadian medical schools. This model promotes the inclusion of diverse stakeholders—such as health professionals, policymakers, academics, and community representatives—ensuring a comprehensive and balanced representation of expertise relevant to the concept of humanism in health care.

#### Eligibility and Participation Criteria

Participant recruitment for this phase will be based on the following eligibility criteria:

- Be aged ≥18 years
- Possess expertise in ≥1 of the following domains: research, health care management, education or training, or community organization
- Demonstrate an interest in the concept of humanism in health care
- Have a good understanding of written French
- Have access to a computer

#### Strategies for Recruiting Experts

An email invitation outlining the purpose of the study and the nature of participation will be sent to experts identified by the research team. This email will include a poster containing a hyperlink and QR code directing recipients to a short expression-of-interest questionnaire. Experts who complete the questionnaire will be contacted by a member of the research team and provided with a more detailed presentation of the project. Those who express interest will then receive, via email, the information and consent form along with a sociodemographic questionnaire, through which they will confirm their participation. Basic demographic information will also be collected to provide an overall profile of panelists and to give an indication of their representativeness (eg, area of expertise, qualifications, experience, country of residence, and gender).

#### Data Collection and Analysis

##### Overview

Each e-Delphi round will span approximately 4 to 6 weeks. At the launch of each e-Delphi round, the survey home page will include a reminder of the method’s objectives, its sequential stages, and the current round’s position within the process. Before accessing the questionnaire, participants will be informed that their responses will be anonymous.

##### Round 1

In the first round of the e-Delphi process, participants will be asked to rate the importance of each pre-established statement using a 4-point Likert scale (1=“Not a priority,” 2=“Low priority,” 3=“Rather a priority,” and 4=“High priority”). Each statement will be accompanied by a brief conceptual rationale to support informed evaluation. Panelists will also be invited to suggest modifications to proposed statements, formulate additional statements, and provide qualitative input through free-text responses. Participants will have 2 weeks to complete the survey and may revise their responses at any time until the survey closes. The estimated completion time is 30 to 45 minutes.

Upon survey closure, descriptive statistics will be used to summarize panelists’ characteristics (eg, professional role, discipline, and years of experience) and Delphi response data during each round. Specifically, for each statement, we will analyze measures of central tendency (median) and dispersion (IQR), as well as the distribution of ratings across response categories. These methods are appropriate given the ordinal nature of Delphi rating scales and are widely recommended for consensus-building studies. Therefore, descriptive statistics will be used to assess the level of agreement; identify areas of convergence or divergence among experts; and guide decisions regarding the retention, modification, or elimination of statements across Delphi rounds. Qualitative comments provided by panelists will complement the quantitative summaries to support the interpretation and refinement of statements. Consensus will be defined a priori as ≥70% of participants rating a statement as either “Rather a priority” or “High priority.” Qualitative data from free-text responses will be thematically analyzed and narratively summarized to enrich the interpretation of the quantitative results by providing contextual depth and insight into expert perspectives. Any newly suggested statements or modifications will be discussed among the research team to determine whether they are relevant for inclusion in the following round.

##### Round 2

Following completion of round 1, anonymized narrative summaries will be shared with all panel members to foster continued engagement, a sense of ownership, and collaborative partnership between participants and the research team. Panelists will receive their individual responses from the first round and the group-level median and IQR for each item.

Participants will be invited to reevaluate and rerate each statement using the same 4-point Likert scale. Additionally, they will be asked to assess any newly proposed or reformulated statements that emerged from round 1.

Descriptive statistical analyses will be conducted to assess consensus using the same criteria described previously: a statement will be considered to have reached consensus if ≥70% of participants rate it as either “Rather a priority” or “High priority.” Qualitative data from open-ended responses will be analyzed and summarized narratively. The research team will review all new suggestions to determine their relevance for inclusion in subsequent rounds.

#### Consensus Meeting

Following the completion of the second round of the e-Delphi survey, a virtual consensus meeting will be held with members of the research team. This meeting will use a nominal group technique to collectively review the results and determine whether a third e-Delphi round is warranted. If a third round is deemed necessary, the same procedures and criteria used in previous rounds will be applied. At the final consensus meeting, the research team will deliberate on and finalize two key outcomes: (1) a conceptual definition of humanism, representing an abstract articulation of its meaning; and (2) an operational definition and a validated set of statements describing how humanism is manifested, taught, and assessed in both academic curricula and health care practice.

### Ethical Considerations

Scientific and ethics approval for this study was obtained on May 12, 2025 (Project #2025-4885). This study will be conducted in accordance with the Tri-Council Policy Statement: Ethical Conduct for Research Involving Humans [34]. The information and consent form will clearly outline the ethical safeguards in place, including confidentiality measures, data retention protocols, and procedures for secure data destruction. All research documents, including raw and processed data, will be stored on secure servers hosted by *Université de Sherbrooke* and protected by dual authentication. Access will be strictly limited to authorized members of the research team. Data will be retained for a period of 7 years following study completion, after which they will be permanently destroyed in accordance with institutional policy. Participants will be clearly informed that their involvement in the study is entirely voluntary and that they are free to withdraw at any point without facing any consequences or having to provide justification. However, in phase 1 (focus groups), participants will be advised that data collected prior to withdrawal cannot be removed, as these data are embedded within collective dialogue and inseparable from group interactions. Removing such data could compromise the scientific integrity of the findings [35]. To promote participant well-being and minimize potential stress related to technology use, technical guidelines will be provided in advance via email, and real-time support will be offered by trained moderators during focus group sessions [25]. In phase 2 (Delphi method), individual responses will be anonymized to prevent bias and preserve the integrity of the consensus-building process.

## Results

This project is funded by the Research Chair in Compassion Science at the *Université de Sherbrooke*, with additional support from the Office of Social Accountability. The study is currently progressing through phase 1. In step 1 (critical literature review), the initial screening of studies based on titles and abstracts has been completed, and full-text screening is nearing completion. To date, 51 studies have been selected, and data extraction and analysis are underway. In step 2, analysis of data collected from 3 focus groups (N=18) has been completed. The process of converting critical review and focus group findings into Delphi statements is expected to be completed by the end of summer 2026. Phase 2 will consist of a modified e-Delphi process. The launch of this phase, involving the submission of the developed statements to an interdisciplinary and cross-sectoral panel of experts, is scheduled for fall 2026.

## Discussion

### Anticipated Findings

This study is designed to generate a conceptually robust and empirically grounded set of consensus-based statements on humanism in health sciences education and practice. Drawing on the ongoing critical literature review and completed focus groups, and building on the structured Delphi process, the study is expected to produce two complementary outputs:

1. A conceptual definition of humanism in health care, grounded in theory, that will clarify the core values, principles, and conceptual boundaries of the concept within the health sciences
2. An operational definition of humanism that will translate this conceptual foundation into actionable domains, attributes, or dimensions, making the concept applicable to education, assessment, and professional development

By combining critical synthesis, empirical qualitative input, and expert consensus building, this study is expected to move beyond fragmented or discipline-specific interpretations of humanism toward a more integrated and operationalizable understanding.

### Comparison With Prior Work

Previous work on humanism in health care has highlighted its importance for professional identity, patient-provider relationships, and ethical practice but has often been characterized by conceptual variability, inconsistent definitions, and heterogeneous measurement approaches. Many existing studies focus on isolated disciplines or rely on predefined frameworks without systematic expert consensus. In contrast, this study explicitly addresses these limitations through a multimethod design. The critical review enables a reflexive examination of theoretical and methodological trends across disciplines, the focus groups provide practice-based perspectives, and the modified Delphi method offers a structured mechanism for achieving interdisciplinary and cross-sectoral consensus. This approach aligns with and extends prior Delphi-based efforts in health professions education by grounding consensus statements in both critical scholarship and empirical data, rather than expert opinion alone.

### Strengths and Limitations

A key strength of this study lies in its methodological integration. The combination of a critical review, focus groups, and a modified e-Delphi process enhances conceptual rigor and credibility while maintaining flexibility to accommodate disciplinary diversity. The involvement of a cross-sectoral expert panel informed by the Partnership Pentagram Model further strengthens the relevance and applicability of the anticipated findings. These perspectives ensure that the resulting definitions and statements are not only theoretically sound but also coconstructed, contextually grounded, and responsive to multiple stakeholder perspectives in education and health care systems (eg, patients, clinicians, educators, institutions, and communities).

However, several limitations should be acknowledged. As a Delphi-based study, findings will reflect the perspectives of the selected expert panel, which may limit generalizability beyond similar contexts or professional cultures. Additionally, although critical reviews prioritize interpretive depth over exhaustiveness, some relevant perspectives may remain underrepresented despite systematic search strategies. Finally, as this manuscript reports on a study in progress, conclusions about consensus outcomes remain prospective and will require confirmation upon completion of the Delphi rounds.

### Future Directions

Once completed, the results of the Delphi study will provide a foundation for multiple next steps. These include empirical testing of how agreed-upon dimensions of humanism translate into educational or clinical outcomes. They may also support the development of educational frameworks or curricular guidelines, as well as the refinement or validation of assessment tools. In addition, future research may examine how the consensus framework operates across cultural or institutional contexts and how it evolves in response to changes in health care systems and professional expectations.

### Dissemination Plan

The findings of this study will be disseminated through multiple, complementary channels to maximize scientific reach, interdisciplinary relevance, and practical impact. First, results from the critical literature review, focus groups, and the modified e-Delphi study will be submitted for publication in peer-reviewed, open-access journals in the fields of health professions education, nursing, and medical education. The use of open-access venues is intended to ensure broad accessibility for researchers, educators, clinicians, and policy-oriented stakeholders. Second, findings will be disseminated through national and international scientific conferences, including conferences focused on health sciences education, humanism in health care, and professional formation. These presentations will allow scholarly exchange, validation, and refinement of interpretations across disciplines. Third, the consensus-based framework developed through the Delphi process will be shared with educational leaders and stakeholders through targeted knowledge translation activities, such as academic seminars, workshops, and invited presentations within health sciences faculties and professional networks. These activities aim to support the integration of findings into educational planning, curriculum development, and assessment initiatives. Finally, the research team will explore opportunities for nontechnical dissemination, including summaries or knowledge briefs tailored for educators, clinicians, and institutional decision-makers, in alignment with the principles of social accountability and knowledge mobilization promoted by the *Université de Sherbrooke*. Together, these dissemination strategies are designed to ensure that the results of this study contribute not only to scholarly knowledge but also to educational practice and future research on humanism in the health sciences.

## Abbreviations

DELPHISTAR: Delphi studies in social and health sciences—recommendations for an interdisciplinary standardized reporting
